# Fecal Microbiota Changes in Patients With Postpartum Depressive Disorder

**DOI:** 10.3389/fcimb.2020.567268

**Published:** 2020-09-29

**Authors:** Yumei Zhou, Chen Chen, Haibo Yu, Zhuoxin Yang

**Affiliations:** The Fourth Clinical Medical College of Guangzhou University of Chinese Medicine, Shenzhen, China

**Keywords:** postpartum depressive disorder, gut microbiota, 16SrRNA gene, gut-brain, sex hormone

## Abstract

Postpartum depressive disorder (PPD) is a unique subtype of major depressive disorder and a substantial contributor to maternal morbidity and mortality. However, the pathogenesis of PPD has still remained elusive, and it may associate with genetic and environmental factors. Gut microbiota has already been proved to be associated with depression; however, a limited number of studies have concentrated on PPD. The present study aimed to explore the potential correlations between gut microbiota and PPD. In this study, 57 participants were enrolled, in which fecal samples of 28 patients with PPD and 16 healthy controls (HCs) were collected and then analyzed by high-throughput sequencing of the 16S ribosomal RNA (rRNA) gene. The results showed that diversity and composition of gut microbial communities were partly different between PPD patients and HCs. The relative abundance of *Firmicutes* phyla was lower in PPD patients. The levels of several predominant genera were significantly different between PPD patients and HCs. More importantly, the PPD patients experienced reduced levels of *Faecalibacterium, Phascolarctobacterium, Butyricicoccus*, and *Lachnospiraceae*, as well as increased levels of *Enterobacteriaceae* family. In addition, a correlation was observed between levels of *Phascolarctobacterium, Lachnospiraceae, Faecalibacterium*, and *Tyzzerella.3* and the severity of depressive symptoms. Various kinds of bacteria, such as *Lachnospiraceae* and *Faecalibacterium*, were found to be associated with levels of sex hormones. This study indicated the correlation between gut microbiota and PPD, and gut microbiota-based biomarkers may be helpful for the diagnosis and treatment of PPD patients. However, further studies need to be conducted to clarify the cause–effect relationship between PPD patients and gut microbiota and to highlight the suitability of gut microbiome as a biomarker.

## Introduction

Postpartum depressive disorder (PPD) is a type of mood disorder associated with childbirth, and it can affect both sexes, associating with the morbidity of 7.1% in the first three postnatal months (Gavin et al., 2005). The symptoms of PPD include extreme sadness, low energy, anxiety, crying episodes, irritability, and changes in sleeping or eating patterns, thereby resembling a major depressive disorder (MDD). PPD severely influences mothers' quality of life and daily activities, and it can also negatively affect the newborn child (Netsi et al., 2018; Weissman, 2018). Despite the fact that multiple antidepressants are recommended for alleviating symptoms of depression, <50% of PPD patients have obvious responses to the existing drug therapies (Hansen et al., 2008). Hormonal abnormalities, neuroendocrine processes, or inflammation all play different roles in depression, although interactions between genetic and environmental factors have significantly attracted scholars' attention. In recent years, mounting evidence has supported the viewpoints that gut microbiome is closely associated with the function and behavior of the brain (Clemente et al., 2012).

The human microbiome, a complex assemblage of the microbes, inhabits, and interacts with human hosts, which are classified into beneficial or pathogenic bacteria. In the human body, great numbers of microbes can be found in the gastrointestinal tract, especially in the colon with the densest and most diverse microbial community (Tremaroli and Backhed, 2012). The gastrointestinal microbiota modulates host function from various aspects: apart from the decomposition of food residues and production of micronutrients, they could produce short-chain fatty acids (SCFAs) (Cummings et al., 1987) and neuroactive substance (Barrett et al., 2012), affect hypothalamic–pituitary–adrenal (HPA) axis (Ait-Belgnaoui et al., 2012) and gut barrier, and promote the balance within the immune system (Clemente et al., 2012).

A number of scholars have supported and characterized “microbiome–gut–brain (MGB) axis” and illuminated a possible role of gut microbiota dysbiosis in a variety of diseases, such as obesity (Torres-Fuentes et al., 2017), inflammatory bowel disease (Lahtinen et al., 2020), and hypertension (Li et al., 2020). Although previous studies have demonstrated that microbial communities may influence our health, further attention needs to be paid to the potential associations between maternal gut microbiome and PPD. However, several studies have suggested that alterations of gut microbiota may influence other depressive disorders. Compared with healthy subjects, the relative abundance of *Bacteroidetes, Proteobacteria*, and *Actinobacteria* has markedly increased in MDD patients, and it was unveiled that *Faecalibacterium* was negatively correlated with the severity of symptoms of depression (Jiang et al., 2015). Another study pointed out that the relative abundance of *Firmicutes, Actinobacteria*, and *Bacteroidetes* has remarkably changed in MDD patients, and fecal microbiota transplantation of Germ-free (GF) mice with “depression microbiota” derived from MDD patients resulted in depression-like behaviors (Zheng et al., 2016). In those studies, the gut microbiota related to inflammatory status, oxidative stress, and disease severity could be further identified (Jiang et al., 2015). Additionally, animal studies showed that changes of gut microbiota were associated with depressive-like behaviors (Foster and McVey Neufeld, 2013), and probiotic supplementation could prevent and alleviate anxiety and depression in mice by regulating gut microbiota dysbiosis (Jang et al., 2019). Besides, a number of scholars have reported the characteristics of maternal microbiome during pregnancy or in the postpartum period. A study assessed the relationship between psychosocial stress and fecal microbiota in pregnant women, and their findings revealed a significant association between anxiety in late pregnancy and women's fecal microbiota composition at the genus level. More specifically, the fecal microbiota of mothers with lower anxiety could be characterized by higher abundance of the *Eubacterium* and *Oscillospira* compared with mothers with higher prenatal anxiety (Hechler et al., 2019). A randomized controlled trial (RCT) demonstrated that targeted supplementation with probiotics can correct PPD and postpartum anxiety-associated behavioral abnormalities (Slykerman et al., 2017). On the basis of these findings, we speculated that gut microbiota dysbiosis may be involved in the development of PPD.

The present study aimed to investigate whether the gut microbiota could be changed in PPD patients and identify the specific microbiota for PPD *via* high-throughput sequencing of the 16S ribosomal RNA (rRNA) gene. Additionally, the associations between gut microbiota and clinical patterns were explored.

## Materials and Methods

### Ethics, Consent, and Permissions

This study was approved by the Ethics Committee of Shenzhen Traditional Chinese Medicine Hospital [Shenzhen, China; Registration No. (2018), 81]. All procedures were designed and conducted in accordance with the Declaration of Helsinki. Eligible participants were informed about all the procedures, benefits, as well as potential risks that they may encounter in this trial, and they could withdraw the study at any time without any specific reason. All the participants signed the written informed consent form prior to commencing the study.

### Recruitment of Study Subjects

Healthy participants and patients with PPD were included in this study. All participants were recruited from the Shenzhen Traditional Chinese Medicine Hospital and Shenzhen Maternity & Child Healthcare Hospital (Shenzhen, China). The PPD was diagnosed according to the Fourth Edition of the Diagnostic and Statistical Manual of Mental Disorders (*DSM-IV*). Participants who gave birth within 1 year were recruited for further evaluation. People were enrolled in this study from June 2019 to October 2019.

The inclusion criteria for patients with PPD were as follows: (1) patients who are aged 20–49 years old; (2) patients who were diagnosed with PPD by a psychiatrist; (3) onset of disease within 12 months after delivery; (4) the scores of 17-item Hamilton depression rating scale (17-HAMD) ranging from 7 to 24; and (5) signing the written informed consent form. Patients with any one of the following items were excluded: (1) bipolar disorder (diagnostic criteria according to *DSM-IV*) or serious mental disorders, such as schizophrenia; (2) dysnoesia or having difficulty in understanding the content of the questionnaire due to brain diseases or other reasons, or incapable of effective interview; (3) pregnancy; (4) the score of “suicide” item in the 17-HAMD would be more than 2; (5) having committed suicide within 1 year; or (6) having anorexia nervosa.

The inclusion criteria for healthy participants were as follows: (1) participants who are aged 20–49 years old; (2) no obvious discomfort; (3) normal health examination after delivery (routine blood count, liver and kidney functions, and electrocardiogram); (4) 17-HAMD score would be <7; and (5) signing the written informed consent form for voluntarily participation in this study. Participants with any of the following conditions were excluded: (1) with pregnancy; (2) having committed suicide in the last year; (3) the score of “suicide” item in 17-HAMD would be >2; or (4) participants who were involved in other clinical trials.

### Evaluation of Clinical Scales

All participants completed the evaluation process of 17-HAMD and the Edinburgh Postnatal Depression Scale (EPDS). The 17-HAMD, a 17-item scale, was designed to measure the frequency and intensity of depressive symptoms in individuals with MDD (Hamilton, 1960). The 17-HAMD scores range from 0 to 52: “7 < scores ≤ 17” indicate clinically mild depression; “17 < scores ≤ 24” are indicative of moderate depression; “scores > 24” denote severe depression. The EPDS, a self-reporting scale, consists of 10 items with acceptable sensitivity, specificity, and positive predictive values (Cox et al., 1987), containing mood, fun, self-accusation, anxiety, fear, insomnia, coping ability, sadness, crying, and self-injury. Each item is divided into four grades: never (0 point), occasionally (1 point), often (2 points), and always (3 points). The total score of EPDS ranges from 0 to 30, and scores higher than 13 are indicative of clinically significant depression.

### Measurement of Serum Levels of Sex Hormones

Blood samples were collected immediately after the 17-HAMD and EPDS assessment, then transferred to the laboratory, and stored in −20°C refrigerator for 15 min until preparation for further analysis. The serum levels of sex hormones, including follicle-stimulating hormone (FSH), luteinizing hormone (LH), prolactinemia (PRL), progesterone (PROG), estradiol (E2), and testosterone (TESTO), were detected using commercially available enzyme-linked immunosorbent assay (ELISA) kits (RayBiotech, Norcross, GA, USA).

### Analysis of Clinical Characteristics

Demographic and clinical characteristics of healthy controls (HCs) and patients with PPD were compared using SPSS 22.0 software (IBM, Armonk, NY, USA). For continuous data, normally distributed data were analyzed by the Student's *t*-test and expressed as mean ± standard deviation, while abnormally distributed data were analyzed by the Mann–Whitney *U*-test and presented as median or interquartile range (IQR). For count data, the chi-square test was used. *P* < 0.05 was considered statistically significant.

### Collection of Fecal Samples

Fecal samples of all participants were collected after being enrolled within 2 days after 17-HAMD and EPDS evaluation, which were put into a sterile plastic cup and immediately stored at −20°C after defecation, and further transported at −80°C storage to the laboratory of Shenzhen Traditional Chinese Medicine Hospital. Additionally, for hospitalized patients, the collected samples were stored in a −80°C refrigerator. The details of fecal sample collection have been previously described (Zhou et al., 2019).

### DNA Extraction

DNA extraction was carried out using MOBIO PowerSoil® DNA Isolation kit, and DNA was stored at −80°C in Tris-EDTA buffer solution. To perform amplification of the V4 region of the 16S rRNA gene and add barcode sequences, unique fusion primers were designed based on the universal primer set, 515F (5′-GTGYCAGCMGCCGCGGTAA-3′) and 806R (5′-GGACTACNVGGGTWTCTAAT-3′), along with barcode sequences. PCR mixtures contained 1 μl of each forward and reverse primer (10 μM), 1 μl of template DNA, 4 μl of dNTPs (2.5 mM), 5 μl of 10 × EasyPfu Buffer, 1 μl of Easy Pfu DNA Polymerase (2.5 U/μl), and 1 μl of double-distilled water in a 50-μl reaction volume. Thermal cycling consisted of an initial denaturation step at 95°C for 5 min, followed by 30 cycles of denaturation at 94°C for 30 s, annealing at 60°C for 30 s, and extension at 72°C for 40 s, with a final extension step at 72°C for 4 min. Amplicons were run for each sample on an agarose gel. Expected band size for 515f-806r was ~300–350 bp. Amplicons were quantified with Quant-iT PicoGreen dsDNA Assay Kit (P11496; Thermo Fisher Scientific, Waltham, MA, USA) according to manufacturer's instructions. The amplicon library for high-throughput sequencing on the Illumina MiSeq V3 reagent PE150 (300 cycles) platform was combined to an equal amount and subsequently quantified using KAPA Library Quantification Kit (KK4824; Illumina, Inc., San Diego, CA, USA) according to manufacturer's protocols.

### High-Throughput Sequencing of 16S Ribosomal RNA Gene

Using the Quantitative Insights Into Microbial Ecology (QIIME) 2.0, the raw sequences were processed to concatenate reads into tags according to the overlapping relationship; then, reads belonging to each sample were separated with barcodes, and low-quality reads were removed. The processed tags were clustered into the Amplicon Sequence Variants (ASVs) at the commonly used 97% similarity threshold. The ASVs were assigned to taxa by matching to the SILVA database. A phylogenetic tree of representative sequences was constructed. Evenness, observed species, Shannon, and Faith-PD indices were used to estimate the α-diversity. The measurement of β-diversity was undertaken using UniFrac that is a β-diversity measure that uses phylogenetic information, and the principal coordinate analysis (PCoA) was employed to calculate the distance matrixes. To further identify the specific bacteria as biomarkers at the genus level, linear discriminant analysis effect size (LEfSe) was applied through the Huttenhower Lab Galaxy Server (Segata et al., 2011) after taxa summaries were reformatted. In the setting of LEfSe, firstly, the Kruskal–Wallis test (α = 0.05) was employed to detect taxa using differential abundance analysis; secondly, the Wilcoxon rank-sum test was used to investigate the biological consistency among subclasses; finally, the effect size of differentially abundant genera was estimated by linear discriminant analysis (LDA) (Segata et al., 2011), and the threshold on the logarithmic LDA score for discriminative features was 2. All the analyses were conducted using “vegan” package in R 3.4.1 software. Correlations between clinical variables and bacterial taxa were analyzed by using Spearman's Rho test.

## Results

### Participants' Demographic and Clinical Data

**Figure 1:**
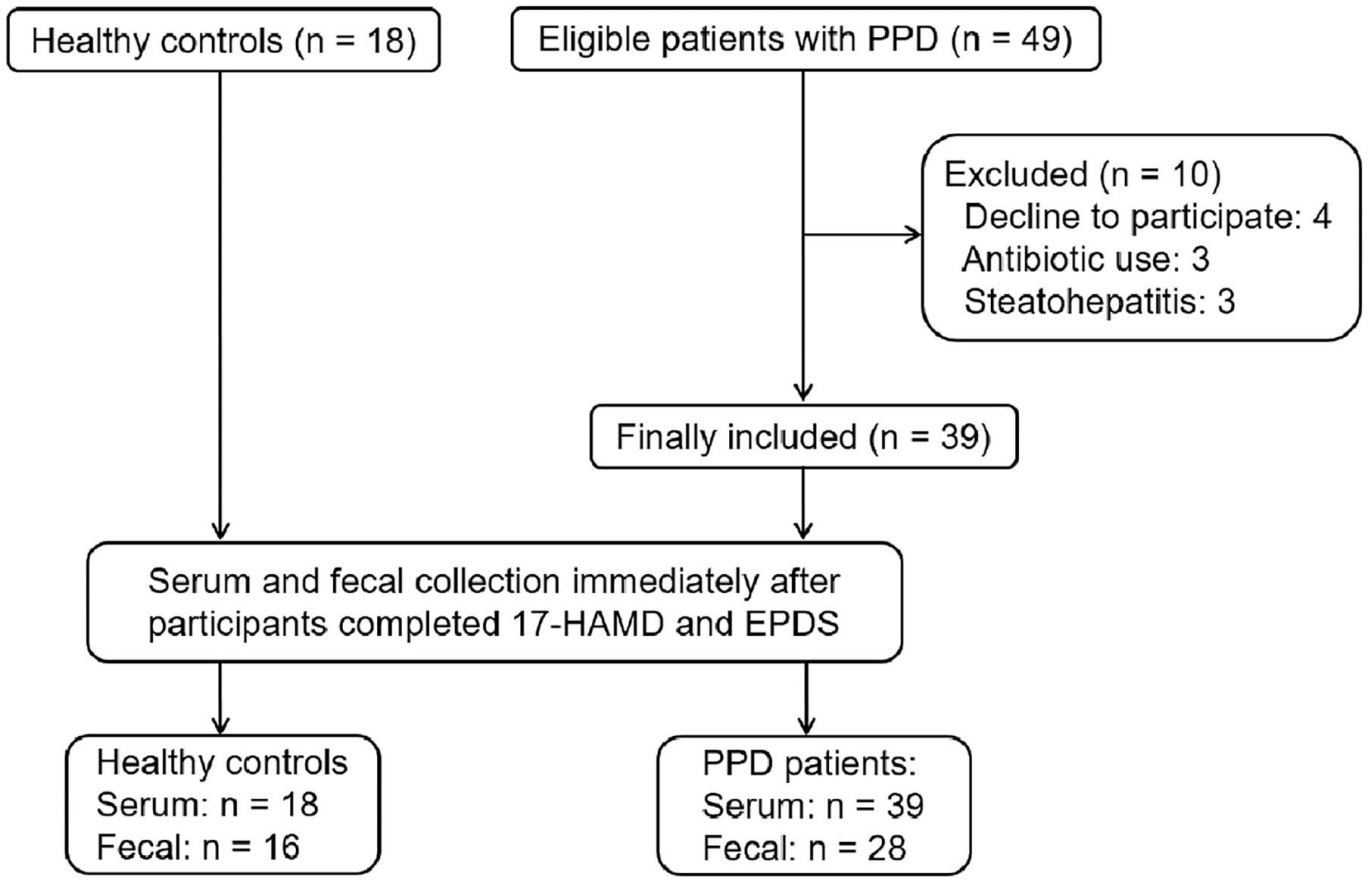
The recruitment of participants and the process of sample collection.

From June 7, 2019 to October 15, 2019, 67 participants were recruited from Shenzhen Traditional Chinese Medicine Hospital and Shenzhen Maternity & Child Healthcare Hospital. Of the 67 participants, 10 patients withdrew from the study. Finally, 18 HCs and 39 patients with PPD were included in the study. All participants completed the 17-HAMD and EPDS evaluation. Among HCs, 18 participants completed the serum sample collection; however, two cases did not complete the fecal sample collection. Among patients with PPD, 39 patients completed the serum sample collection, and only 28 cases completed the fecal sample collection (Figure 1).

Demographic characteristics, such as age, body mass index (BMI), education background, duration of postpartum, etc., showed no significant difference between the HC group and PPD group (Table 1; *P* > 0.05). For the EPDS and 17-HAMD, the total score was significantly higher in the PPD group than that in the HC group (Table 1; *P* < 0.001). Other baseline information was also collected, including special diets, gastrointestinal disorders, metabolic disorders, and antibiotic/probiotic treatments. The surveys showed that all puerpera had no irritable bowel syndrome and bowel cancer; however, some of them had constipation (Table 1). None had hypertension or diabetes after childbirth, while three participants were excluded for steatohepatitis (Figure 1). Additionally, another three patients with antibiotic use were ruled out as well (Figure 1). Furthermore, all the participants were from Shenzhen (China), whose favorable diet was easy-to-digest foods and less consumption of greasy, Atsumi, and spicy food. All the eligible participants had a general appetite and nutritious food with balanced portion of vegetables and meat.

**Table 1:** Baseline characteristics and clinical symptoms in patients with PPD and HCs.

**Parameter**	**HC group**	**PPD group**	***P*-value**
	**(*n* = 18)**	**(*n* = 39)**	
**Sociodemographics**			
Age, mean (SD), years	32.57 ± 3.98	33.64 ± 4.27	0.376
BMI, mean (SD)	20.90 ± 2.24	21.50 ± 2.87	0.434
High school or less, No. (%)	2 (11.11%)	7 (17.95%)	0.275
Duration of postpartum, mean	142.94 ± 129.00	112.00 ± 91.84	0.372
(SD), days			
Menstruating, No. (%)	11 (61.1%)	30 (76.0%)	0.217
Constipation, No. (%)	5 (27.8%)	16 (41.0%)	0.335
**Severity of depressive symptoms**			
HAMDs	3.83 ± 1.98	13.46 ± 3.51	<0.001*
EPDS	5.72 ± 3.72	15.33 ± 4.66	<0.001*

HC, healthy control; PPD, postpartum depressive disorder; HAMDS, Hamilton's Depression Scale; EPDS, Edinburgh Postnatal Depression Scale. P < 0.05 is marked with *.

The serum levels of sex hormones in the PPD group were compared with those in the HC group (Table 2). No significant differences in the levels of FSH, LH, and PROG were observed between the two groups. However, the levels of E2 and TESTO in the PPD group were lower than those in the HC group (*P* = 0.036 and 0.012, respectively), and the PRL level in the PPD group was higher than that in the HC group (*P* = 0.001).

**Table 2:** Sex hormone levels in patients with PPD and HCs.

**Parameter**	**HCs group**	**PPD group**	***P*-value**
	**(*n* = 18)**	**(*n* = 39)**	
FSH, mean (SD), ng/ml	9.05 ± 5.74	8.59 ± 3.01	0.757
LH, mean (SD), ng/ml	14.73.90 ± 28.25	6.04 ± 5.26	0.212
E2, mean (SD), ng/ml	240.61 ± 331.17	109.42 ± 125.33	0.036*
PRL, mean (SD), ng/ml	346.43 ± 265.54	872.82 ± 860.03	0.001*
PROG, mean (SD), ng/ml	4.86 ± 13.49	2.005 ± 4.41	0.392
TESTO, mean (SD), ng/ml	1.27 ± 0.61	0.82 ± 0.612	0.012*

HC, healthy control; PPD, postpartum depressive disorder; FSH, follicle-stimulating hormone; LH: luteinizing hormone; E2, estradiol; PRL, prolactinemia; PROG, progesterone; TESTO, testosterone. P < 0.05 is marked with *.

### Collection of 16S Ribosomal RNA Sequences

Herein, 44 samples from participants who completed 16S rRNA sequences were collected, in which 1,765,950 qualified sequences from 1,852,840 raw sequences were filtered. Then, a total of 979 qualified ASVs were clustered for downstream analysis. The mean number of ASVs per sample was 92.70, and the standard deviation was 23.86. The sequencing results could be achieved from 28 PPD patients and 16 HCs.

### Analysis of Microbial α- and β-Diversity

**Figure 2:**
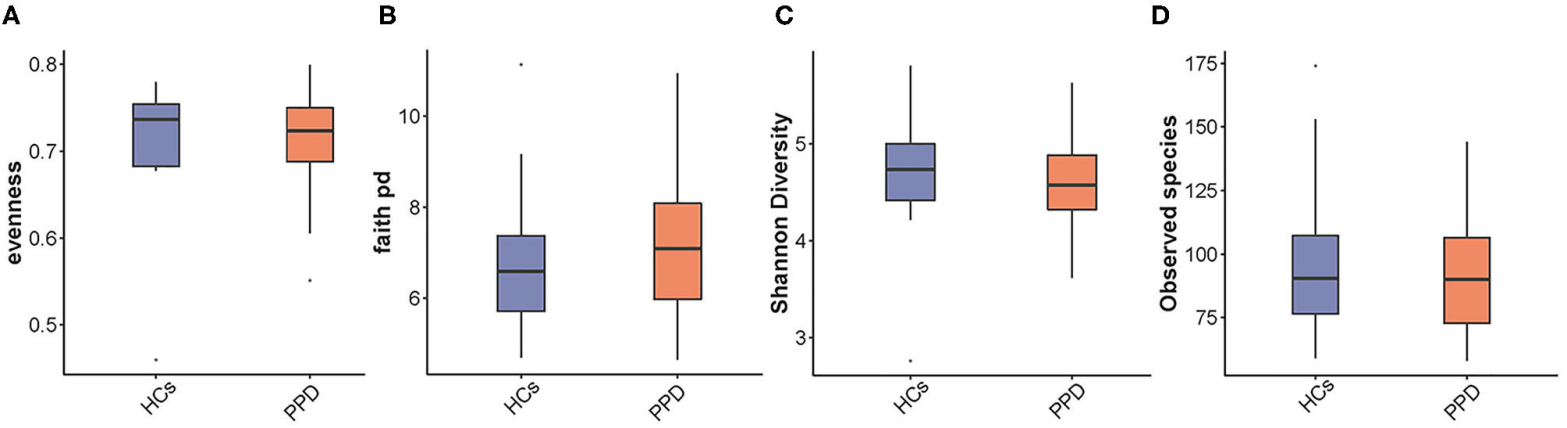
An analysis of microbial α-diversity. Evenness index **(A)**; Faith pd index **(B)**; Shannon diversity **(C)**; Observed species **(D)**.

The indices of fecal bacterial α-diversity are shown in Figure 2. There were no significant differences between the PPD group and HC group, including observed species, Evenness, Shannon, and Faith-PD indices (*P* = 0.669, 0.526, 0.367, and 0.435, respectively) (Figure 2). However, the observed species seemed to be much higher in the HC group than those in the PPD group (Figure 2D).

**Figure 3:**
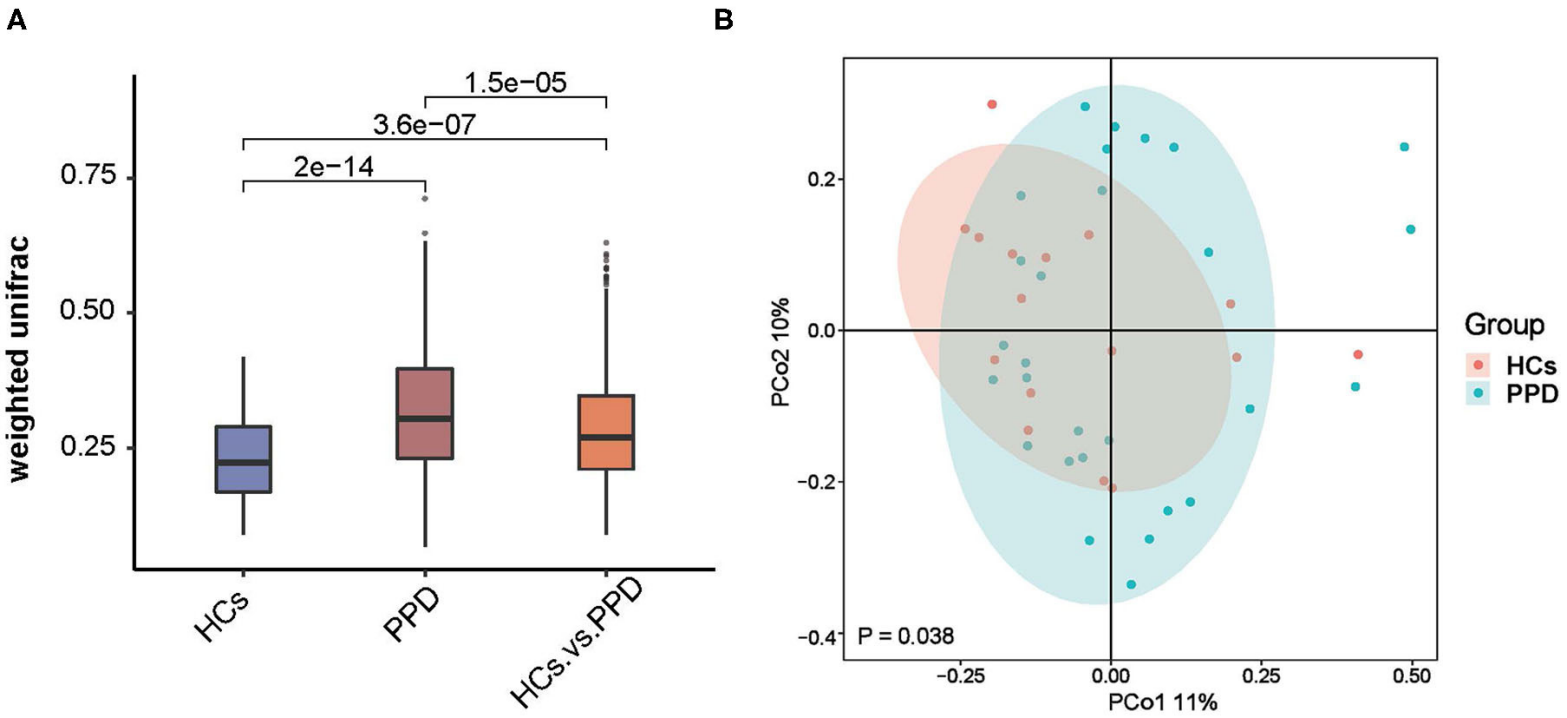
An analysis of microbial β-diversity. The Wilcoxon rank-sum test analysis **(A)** and Principal coordinates analysis plots **(B)** of the fecal microbiome based on the weighted-UniFrac distance metric.

The indices of fecal bacterial β-diversity are illustrated in Figure 3. The Wilcoxon rank-sum test and PCoA were performed to measure differences in β-diversity between the two groups using weighted UniFrac distance metrics (Figures 3A,B, respectively). The sample-based differences in the PPD group were significantly higher than those in the HC group (*P* = 2e-14) (Figure 3A). Additionally, as displayed in Figure 3B, the result of PCoA unveiled that there was a significant difference in bacterial communities between the PPD group and HC group (*P* = 0.038). The results disclosed that the indices of fecal bacterial β-diversity in the HC group were more centralized than those in the PPD group.

### Composition of Microbial Communities

**Figure 4:**
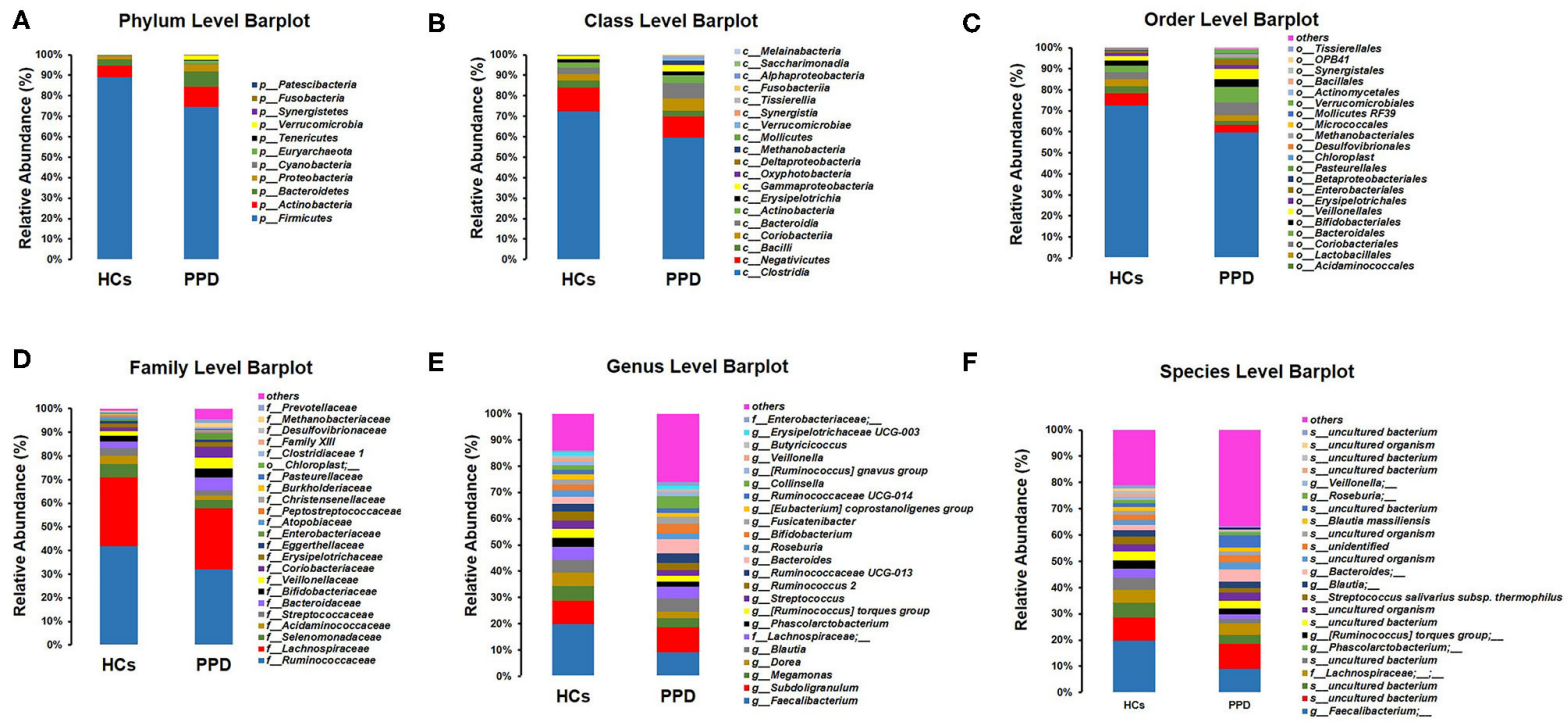
Composition of microbial communities. Phylum level **(A)**; Class level **(B)**; Order level **(C)**; Family level **(D)**; Genus level **(E)**; Species level **(F)**.

The histograms of species were made for the two groups at the levels of phylum, class, order, family, and genus on the basis of the annotation results. The histograms showing the relative abundance of species uncovered the composition (species and corresponding proportion) of microbial communities in each group with higher relative abundance at different levels (Figures 4A–F). The analysis of the composition of the gut microbiota at the levels of phylum and genus reflected the entire structure of gut microbiota.

On the whole, there were significant differences in the composition of the gut microbiota at the phylum level (Figure 4A). Besides, 10 and 11 phyla were detected in the HC group and PPD group by sequence alignment analysis using the SILVA database, respectively. *p_Patescibacteria* was only found in the PPD group, although its relative abundance was low. In both groups, the proportion of *p_Firmicutes* was the highest among all phyla, which was higher in the HC group (88.91%) than that in PPD group (74.57%). *p_Actinobacteria* and *p_Bacteroidetes* ranked the second and third, while they were both relatively lower in the HC group than those in the PPD group.

Furthermore, we analyzed the characteristics and alterations in community structure of the gut microbiota in the two groups at the genus level according to the relative abundance (Figure 4E, Table 3). Among all bacteria, *g_Faecalibacterium* was the most dominant one, accounting for 19.79% in HC group and 9.22% in the PPD group. The prominent changes of community structure of the gut microbiota at the genus level are related to the decrease of *g_Faecalibacterium* (*P* = 0.003), *g_Phascolarctobacterium* (*P* = 0.022), *g_Butyricicoccus* (*P* = 0.024), and *g_Megasphaera* (*P* = 0.047) in the PPD group compared to those in the HC group (Table 3).

**Table 3:** Relative abundance of gut microbial communities at the genus level.

	**Tax_name**	**HCs (%)**	**PPD (%)**	***P*-value**
1	*g_Faecalibacterium*	19.7913	9.2243	0.003
2	*g_Subdoligranulum*	8.9888	9.4907	0.778
3	*g_Megamonas*	5.6413	3.4550	0.366
4	*g_Dorea*	5.0988	2.3821	0.090
5	*g_Blautia*	4.9138	5.1350	0.826
6	*f_Lachnospiraceae;_;_*	4.7738	4.3886	0.534
7	*g_Phascolarctobacterium*	3.4688	1.9243	0.022
8	*g_[Ruminococcus] torques group*	3.3713	2.3286	0.163
9	*g_Streptococcus*	3.3200	2.0829	0.227
10	*g_Ruminococcus 2*	3.2888	2.7879	0.864
11	*g_Ruminococcaceae UCG-013*	2.9538	3.5471	0.788
12	*g_Bacteroides*	2.6638	5.5364	0.714
13	*g_Roseburia*	2.4225	2.0914	0.485
14	*g_Bifidobacterium*	2.3725	3.6564	0.288
15	*g_Fusicatenibacter*	1.9988	2.8257	0.668
16	*g_[Eubacterium] coprostanoligenes group*	1.8713	1.4979	0.837
17	*g_Ruminococcaceae UCG-014*	1.7888	1.6050	0.598
18	*g_Collinsella*	1.5913	4.5729	0.225
19	*g_[Ruminococcus] gnavus group*	1.5125	1.4343	0.882
20	*g_Veillonella*	1.1950	0.5864	0.193
21	*g_Butyricicoccus*	1.0888	0.6300	0.024
22	*g_Erysipelotrichaceae UCG-003*	1.0800	1.2386	0.892
23	*f_Enterobacteriaceae;_*	0.6763	1.5679	0.712
24	*g_Megasphaera*	0.6125	0.4636	0.047
25	*g_Anaerostipes*	0.6025	0.7200	0.345
26	*g_Tyzzerella 4*	0.5763	0.4671	0.516
27	*g_Adlercreutzia*	0.5600	0.2793	0.221
28	*g_Olsenella*	0.5200	0.1986	0.972
29	*Others*	11.2575	23.8821	

*HC, healthy control; PPD, postpartum depressive disorder*.

### Comparing Differences in Bacterial Genus Between the Healthy Control Group and Postpartum Depressive Disorder Group

**Figure 5:**
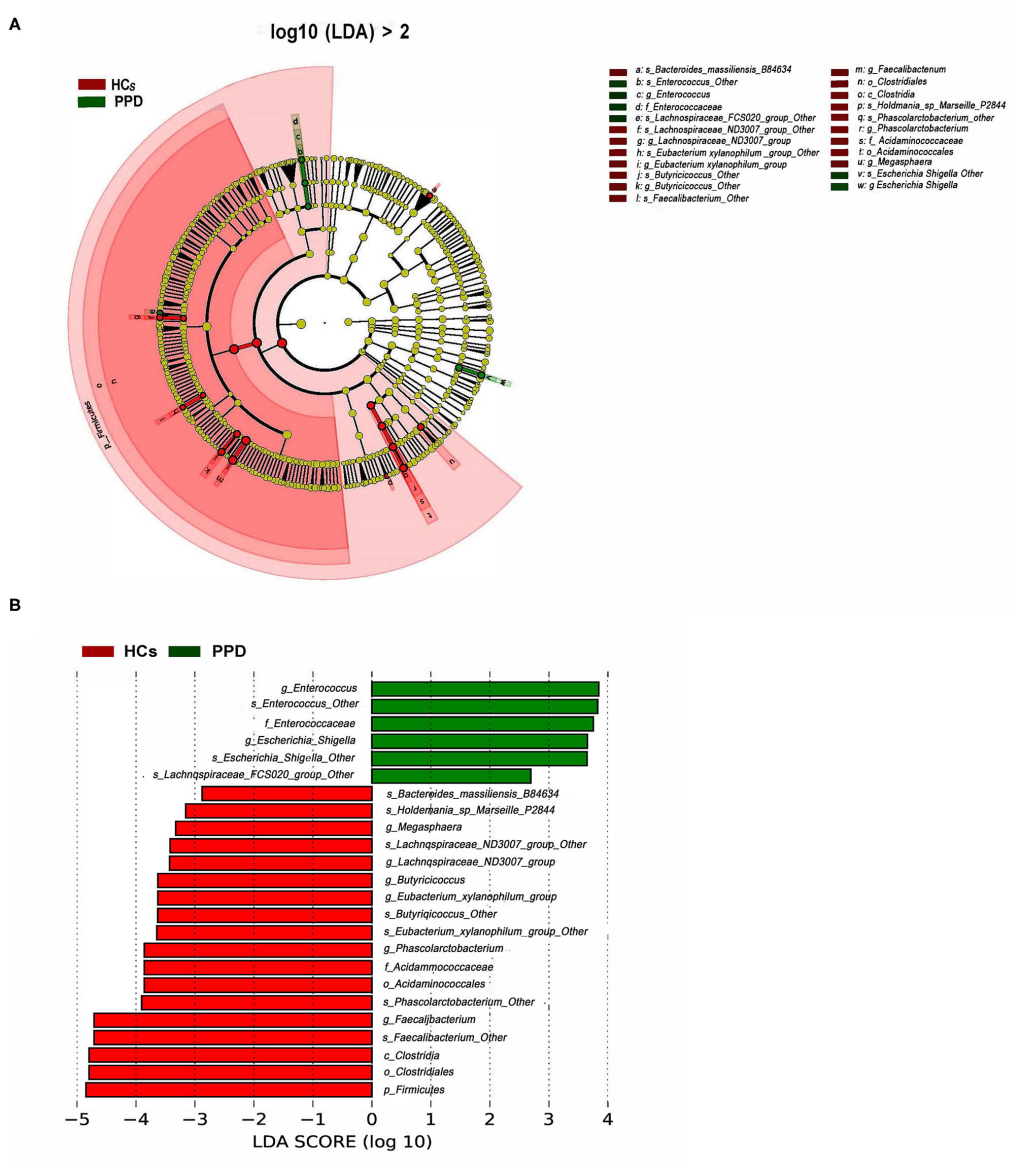
Bacterial taxa differences between healthy control (HC) and postpartum depressive disorder (PPD) patient samples. Cladogram showing the most differentially abundant taxa identified by linear discriminant analysis effect size (LEfSe). Red indicates clades enriched in the HC group, whereas blue indicates clades enriched in the PPD group **(A)**. Comparisons of gut microbiota between the NC and PPD groups **(B)**. Only genera meeting a linear discriminant analysis score threshold >2 are shown.

To further investigate the differences in the abundance between the two groups and to explore the specific bacteria associated with PPD, LEfSe analysis was utilized (*P* < 0.05, LDA value > 2). The most significant difference among the two groups was that *Faecalibacterium, Phascolarctobacterium, Butyricicoccus, Lachnospiraceae, Acidaminococcaceae, Eubacterium_xylanophilum*, and *Megasphaera* were mainly enriched in the HC group, and *Enterocossus* and *Escherichia_Shigella* were mainly enriched in the PPD group (Figures 5A,B).

### Associations of Gut Microbiota With Clinical Indicators and Sex Hormones

#### Associations of Gut Microbiota With Clinical Indicators

BMI was positively correlated with *g_Allisonella* but negatively correlated with *g_Holdemania, g_Coprobacillus*, and *g_Ruminococcaceae.UCG.014*. Age was positively correlated with *g_Allisonella, g_Raoultibacter*, and *g_Fournierella*, while that was negatively correlated with *g_Moryella* and *g_Megasphaera*.

**Figure 6:**
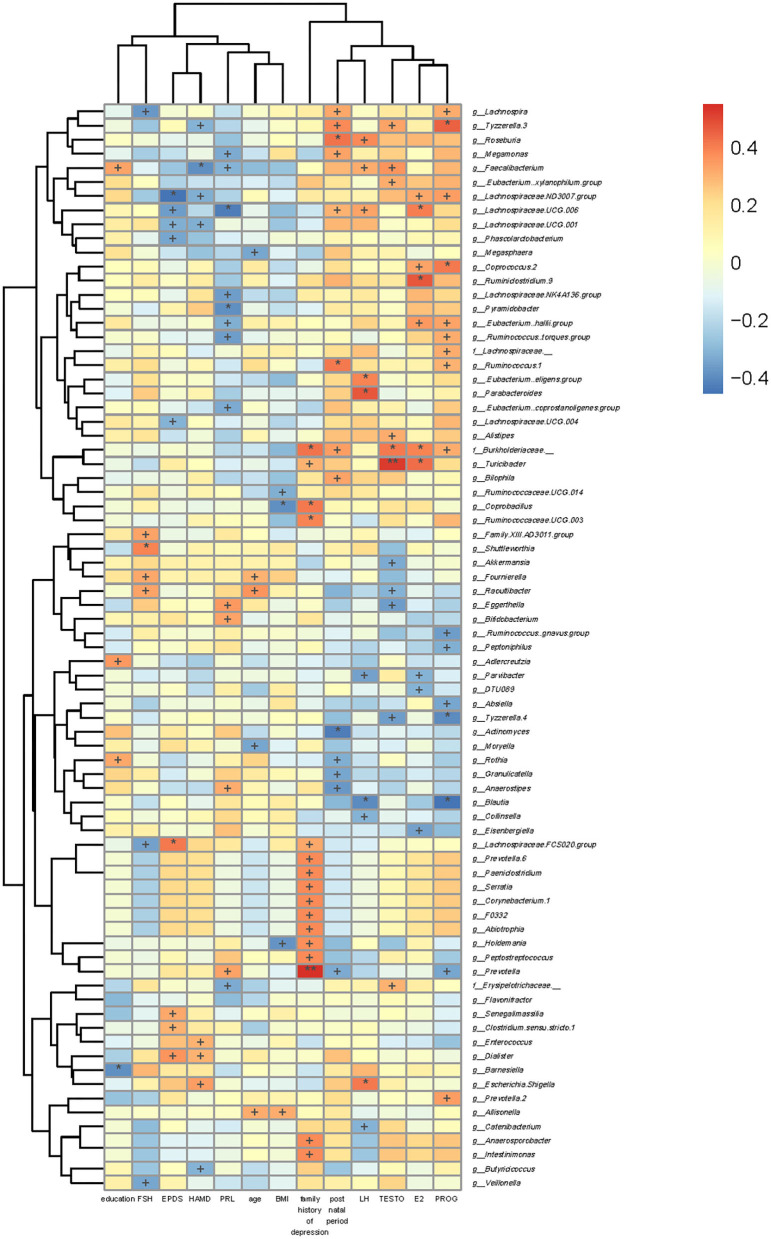
Association of gut microbiota with clinical indicators. The heat map of Spearman's rank correlation coefficients between the gut microbiota and clinical indicators. ^+^*P* < 0.05; *P < 0.01; **P < 0.001.

EPDS scores were positively correlated with *g_Dialister, g_Clostridium.sensu.stricto.1, g_Senegalimassilia*, and *g_Lachnospiraceae.FCS020.group*, while those were negatively correlated with *g_Lachnospiraceae.UCG.004, g_Phascolarctobacterium, g_Lachnospiraceae.UCG.001, g_Lachnospiraceae.UCG.006*, and *g_Lachnospiraceae.ND3007.group*. The 17-HAMD scores were positively correlated with *g_Escherichia.Shigella, g_Dialister*, and *g_Enterococcus*, while those were negatively correlated with *g_Butyricicoccus, g_Lachnospiraceae.UCG.001, g_Lachnospiraceae.ND3007.group, g_Faecalibacterium*, and *g_Tyzzerella.3* (Figure 6).

### Associations of Gut Microbiota With Levels of Sex Hormones

We also analyzed the associations between levels of sex hormones and gut microbiota. FSH level was positively correlated with *g_Raoultibacter, g_Fournierella, g_Shuttleworthia*, and *g_Family.XIII.AD3011.group*, while that was negatively correlated with *g_Veillonella, g_Lachnospiraceae.FCS020.group*, and *g_Lachnospira*. Moreover, PRL level was negatively correlated with *f_Erysipelotrichaceae_*,*g_Eubacterium..coprostanoligenes.group, g_Ruminococcus..torques.group, g_Lachnospiraceae.NK4A136.group, g_Pyramidobacter, g_Eubacterium..hallii.group, g_Lachnospiraceae.UCG.006, g_Megamonas*, and *g_Faecalibacterium*, while that was positively correlated with *g_Prevotella, g_Anaerostipes, g_Bifidobacterium*, and *g_Eggerthella*. PROG level was positively correlated with *g_Prevotella.2, f_Burkholderiaceae._, g_.Eubacterium..hallii.group, g_.Ruminococcus..torques.group, f_Lachnospiraceae._, g_Ruminococcus.1, g_Coprococcus.2, g_Lachnospiraceae.ND3007.group, g_Lachnospira*, and *g_Tyzzerella.3*, whereas that was negatively correlated with *g_Prevotella, g_Blautia, g_Absiella, g_Tyzzerella.4, g_.Ruminococcus..gnavus.group*, and *g_Peptoniphilus*. TESTO level was positively correlated with *f_Erysipelotrichaceae._, g_Alistipes, f_Burkholderiaceae._, g_Turicibacter, g_Faecalibacterium, g_.Eubacterium.xylanophilum.group*, and *g_Tyzzerella.3*, while it was negatively correlated with *g_Tyzzerella.4, g_Raoultibacter, g_Eggerthella*, and *g_Akkermansia*. LH level was negatively correlated with *g_Catenibacterium, g_Blautia, g_Collinsella, g_Parvibacter, g_Lachnospiraceae.UCG.006, g_Faecalibacterium*, and *g_Roseburia*, while that was positively correlated with *g_Escherichia.Shigella, g_.Eubacterium..eligens.group*, and *g_Parabacteroides*. E2 level was *g_Eisenbergiella, g_Parvibacter*, and *g_DTU089*, while that was positively correlated with *f_Burkholderiaceae._, g_Turicibacter*, g*_.Eubacterium..hallii.group, g_Coprococcus.2, g_Ruminiclostridium.9, g_Lachnospiraceae.ND3007.group*, and *g_Lachnospiraceae.UCG.006* (Figure 6).

## Discussion

In the current study, we characterized the gut microbiota of PPD and HC patients by high-throughput sequencing of the 16S rRNA gene. Moreover, associations of gut microbiota with clinical indicators and sex hormones were assessed. The results showed that diversity and composition of gut microbial communities were remarkably different between the PPD group and HC group. We found that some butyrate-producing genera were enriched in the HC group, such as *Faecalibacterium, Phascolarctobacterium*, and *Butyricicoccus*. Additionally, the overgrowth of *Enterococcaceae* and *Escherichia_Shigella* was detected in the PPD group. Moreover, the present study revealed that the amounts of specific genera were correlated with clinical indicators and levels of sex hormones in the PPD group, which indicated that sex hormones might play a significant role in the gut microbiome of patients with PPD.

Additionally, no significant difference in α-diversity was noted between the PPD group and HC group. Similarly, a recent research revealed that there were no significant differences in the Shannon's index and Simpson's index between patients with MDD and non-depressed controls (Sanada et al., 2020). However, β-diversity of gut microbiota, referring to the sample-based differences, was markedly greater in the PPD group than those in the control group. Various factors may influence the diversity of gut microbiota, including age, diet, and health status, and a number of studies suggested that keeping a rich diversity of gut microbiota is highly beneficial for protecting from autoimmune and metabolic diseases (Manichanh et al., 2006; Claesson et al., 2012; Le Chatelier et al., 2013). However, other studies demonstrated that microbial diversity is reduced in patients with MDD (Dantzer et al., 2018; Winter et al., 2018). Hu et al. (2019) found that diversity of gut microbiota was noticeably different between patients with bipolar disorders and HCs. Another one studied the interaction between antidepression treatments and gut microbiota in a mouse model of depression and found an obvious increase of species richness in acupuncture-treated mice, which indicated that the diversity of the gut microbiota may be a factor influencing depression (Fu, 2019). Thus, due to controversial results about bacterial diversity, further research is warranted to explore the differences in bacterial α- and β-diversity between patients with PPD and HCs.

The present study demonstrated that the microbial composition in the PPD group was noticeably different from that in the HC group. Phylum *Firmicutes* ranked the first in both groups, and it was markedly lower in the PPD group than that in the HC group, which was consistent with findings of a previous research (Hu et al., 2019). Additionally, we found that *Escherichia_Shigella* was enriched in the PPD group. *Escherichia_Shigella*, belonging to the *Enterobacteriaceae* family, is Gram-negative bacteria and observed in normal gut flora. Overgrowth of *Enterbacteriaceae* could result in gut inflammation and increased permeability of the gut wall, which in turn favors bacterial translocation, promoting systemic inflammation (Winter and Bäumler, 2014). Clinical depression is accompanied by increased pro-inflammatory cytokine interleukin (IL), such as IL-1β and IL-6 (Berk et al., 2013; Wong et al., 2016). Some studies found an anti-inflammatory effect of *Faecalibacterium, Bifidobacterium*, and *Lactobacillus* on stress responses and depressive disorders (Jiang et al., 2015; Aizawa et al., 2016). Moreover, previous studies have also found that the increase of *Enterobacteriaceae* in the gastrointestinal tract can induce behavioral and psychological changes in animals and humans (Goehler et al., 2008; Löwe et al., 2014; Jiang et al., 2015; Borgo et al., 2017). More importantly, various bacteria with decreased abundance were found in the PPD group, including *Faecalibacterium, Phascolarctobacterium*, and *Butyricicoccus*. It was in accordance with some studies (Jiang et al., 2015; Zheng et al., 2016) that reported lower abundance of *Faecalibacterium* in depressive patients compared with non-depressed individuals, while the study by Chen et al. (2018) presented a contrary result. *Faecalibacterium* is a genus of bacteria, and *Faecalibacterium prausnitzii*, its only known species, produces butyric acid and other SCFAs (Louis and Flint, 2009). In addition, a recent study demonstrated that the intake of *Faecalibacterium prausnitzii* improves anxiety-related and depressive-like behaviors in the preclinical setting (Hao et al., 2019), and the study by Jiang et al. (2015) showed a negative correlation between the abundance of *Faecalibacterium* and the severity of depression symptoms. Besides, *Phascolarctobacterium*, affiliated with the *Acidaminococcaceae* at the family level, was significantly higher in the HC group, which is in line with a study that assessed and compared patients with MDD with non-depressed controls (Jiang et al., 2015). However, another study (Jeffery et al., 2012) concentrated on patients with both irritable bowel syndrome and depression and found that *Acidaminococcaceae* significantly overgrew using pyrosequencing fecal samples. Additionally, we also noted lower abundance of *Butyricicoccus* at the genus level in the PPD group. *Butyricicoccus* belongs to the *clostridial* cluster IV genus of the *Firmicutes* phylum, which is typically decreased in patients with inflammatory bowel disease (Eeckhaut et al., 2013). The inflammatory bowel conditions have been found to be correlated with high comorbidity with depression and anxiety (Bhandari et al., 2017). Moreover, we found that the abundance of *Butyricicoccus* was positively associated with EPDS scores. *Butyricicoccus pullicaecorum* is an anaerobic and butyrate-producing bacterium from the genus *Butyricicoccus*. Butyrate, one of the main products of colonic microbiota, has been found beneficial for a variety of diseases, such as insulin resistance and ischemic stroke (Canani et al., 2011). Furthermore, butyrate in the central nervous system can influence the function of the hippocampus and promote the expression of brain-derived neurotrophic factor (BDNF), which has been shown to have antidepressant-like effects in animal models (Yamawaki et al., 2012; Wei et al., 2014). Thus, decreased butyrate-producing bacteria in PPD patients may contribute to the disease pathology.

Furtherly, we noted that the gut microbial communities were relevant to confounding factors, including clinical indicators (age, BMI, etc.), disease severity, and levels of sex hormones. Among the PPD patients, BMI was found to be positively correlated with *g_Allisonella* but negatively correlated with *g_Holdemania, g_Coprobacillus*, and *g_Ruminococcaceae.UCG.014*. In another study that involved patients with bipolar disorders, *Holdemania* at the genus level was also found to be negatively correlated with BMI (Hu et al., 2019). Another study showed that the abundance of *Ruminococcaceae* increased in obesity patients (Chávez-Carbajal et al., 2019), and *Ruminococcaceae* was associated with antidepressant effects in rats under chronic mild stress (Tung et al., 2019). Moreover, the increased levels of *Ruminococcaceae* would decrease after weight reduction (Kang et al., 2017). *Ruminococcaceae* is a dominant butyrate producer. Butyrate supports the energy for colonic mucosa and makes a different regulatory effect on gene expression and inflammation (Pajak et al., 2007; Hamer et al., 2008; Ivanov and Honda, 2012). These findings indicated that *Ruminococcaceae* may be related to the development of PPD through metabolic pathways. Besides, *Holdemania* at the genus level is involved in glucose metabolism and metabolic syndrome (Lippert et al., 2017).

Previous studies have reported some associations between the levels of several genera and depression severity in MDD (Naseribafrouei et al., 2014; Jiang et al., 2015). In the present study, EPDS scores were negatively associated with *Phascolarctobacterium* and *Lachnospiraceae* at the genus level, and 17-HAMD scores were positively associated with *Escherichia.Shigella, Dialister*, and *Enterococcus* and negatively associated with *Butyricicoccus, Lachnospiraceae, Faecalibacterium*, and *Tyzzerella.3* at the genus level. Hence, gut microbiota may play a pivotal role in the metabolic disturbance in PPD patients.

With respect to the causes of PPD, although it has been poorly understood, a number of human and animal studies supported the role of sex hormones in PPD (Schiller et al., 2015). In the current study, we also found that the serum levels of E2, PROG, and TESTO were noticeably different between the PPD group and HC group. Recently, various studies have reported that changes in the levels of sex hormones could be related to diversity and profiles of gut microbiota (Baker et al., 2017; Shin et al., 2019). The gut microbiota has been shown to be influenced by E2; meanwhile, the gut microbiota also could regulate E2 level through secreting β-glucuronidase (Flores et al., 2012; Huang et al., 2017). In the present research, our results also found that some bacteria genera were associated with serum sex hormone levels in PPD patients, such as *Faecalibacterium, Lachnospiraceae*, and *Megamonas*, which were significantly different from those in HCs. A recent study has demonstrated that the regulation of sex hormones–microbiota–inflammation axis could ameliorate polycystic ovary syndrome (PCOS), including *Faecalibacterium, Parabacteroides, Bifidobacterium*, and so on (Wang et al., 2020). Another study showed that prenatal androgen exposure causes hypertension and gut microbiota dysbiosis (Sherman et al., 2018). Moreover, a study found that small amounts of brain E2 and PROG could improve menopausal symptoms by decreasing serum FSH levels and maintaining the diversity of the gut microbiome in estrogen-deficient rats (Park et al., 2018). Our study used EPDS and 17-HAMD scores to assess symptoms of depression in PPD patients and found that *Faecalibacterium* and *Lachnospiraceae* were correlated with disease severity. Hence, the abovementioned findings showed that the interaction of sex hormones and gut microbiota may play a substantial role in PPD.

## Conclusions

In summary, our findings may provide further evidence to support a number of previous reports that the gut microbial composition of PPD patients partially differs from that of HCs, and we explored new associations among gut microbiota, disease severity, and sex hormones. We also noted that *Faecalibacterium, Phascolarctobacterium*, and *Butyricicoccus* were significantly decreased in patients with PPD, which were all butyrate-producing, as well as *Enterobacteriaceae* family increased obviously. In addition, we demonstrated that *Phascolarctobacterium, Lachnospiraceae, Faecalibacterium*, and *Tyzzerella.3* were correlated with disease severity; besides, various kinds of bacteria, such as *Lachnospiraceae* and *Faecalibacterium*, were found to be associated with the levels of sex hormones. The abovementioned results may assist scholars to further explore the underlying pathogenesis of PPD. Moreover, the identified microbiota in this study could be a potential diagnostic biomarker of PPD. Therefore, our findings may provide significant clues for future researches.

## Limitations

Serval limitations of this study should be pointed out. Firstly, this observational study may not contain reliable indicators of causal effects; thus, further longitudinal studies on patients with depression are required to clarify the cause–effect relationship between PPD and gut microbiota. Secondly, due to the small sample size, we could not adjust for multiple testing or ethnicity, and our results still need to be validated by further studies with larger sample sizes. Therefore, future studies may elucidate the temporal and causal relationships between gut microbiota and PPD.

## Data Availability Statement

The original contributions presented in the study are publicly available. This data can be found here: https://www.ncbi.nlm.nih.gov/, with accession number PRJNA637228.

## Ethics Statement

The studies involving human participants were reviewed and approved by Medical Ethical Review committee of Shenzhen Traditional Chinese Medicine Hospital (Shenzhen, China; Approval No. [2018], 81). The patients/participants provided their written informed consent to participate in this study.

## Author Contributions

YZ conceived and planned the experiments and wrote the manuscript. YZ and CC executed the experiments. HY analyzed the data. ZY contributed to revise the final manuscript. All authors approved the submitted version.

## Conflict of Interest

The authors declare that the research was conducted in the absence of any commercial or financial relationships that could be construed as a potential conflict of interest.
